# Editorial: Insights in exercise and diabetes management

**DOI:** 10.3389/fendo.2022.1009564

**Published:** 2022-09-12

**Authors:** T. Cyriac, B. Haastert, M. E. Francois

**Affiliations:** ^1^ School of Medical, Indigenous and Health Sciences, University of Wollongong, Wollongong, NSW, Australia; ^2^ Illawarra Health and Medical Research Institute, Wollongong, NSW, Australia; ^3^ mediStatistica, Wuppertal, Germany

**Keywords:** physical activity, yoga, insulin pump, continuous glucose, type 1 diabetes

A rise in the prevalence of diabetes mellitus and associated cardiovascular risk is a global concern as the longevity of the population is increasing worldwide. Diabetes and associated complications affect the quality of life of the individual as well as incur additional costs and economic loss to the affected and family, the healthcare system and national economies worldwide (1).

Cost-effective and scalable lifestyle and behavioural interventions are of imperative immediate need to prevent and better manage diabetes, especially when rising trends are being observed in younger populations. This Research Topic highlights several novel strategies which can be used to further exercise management, including yoga, insulin pump and carbohydrate dose algorithms, and medication interactions.

Yoga, a meditative posture-based physical fitness routine with origins in ancient India, is a long-standing evidence-based therapy to improve health and contribute to a reduced risk of type 2 diabetes (T2D). A recent study explores the role of a Yoga-based lifestyle modification trial in prediabetes. The multicentric, pan-Indian RCT by Raghuram et al. aimed to test the effectiveness of Yoga in delaying the onset of T2D in people with prediabetes. Following the 3-month trial, over half of the participants delayed their progression to diabetes compared to standard care. Noteworthy was also the reversal to normoglycaemia in younger participants.

Type 1 Diabetes (T1D) is a challenging diagnosis for the affected, caregivers and the healthcare team. Managing insulin dosage and hypoglycaemic events complicate it further. In children and adolescents, fear of hypoglycaemia (FOH) is common, affecting their daily activities of living and quality of life. Often, FOH prevents families with T1D children from attaining high physical activity levels or engaging in sport. Two articles in this series attempt to navigate through these factors by investigating continuous glucose monitoring, insulin pump systems and carbohydrate dosage to improve exercise management.

Jabbour and Bragazzi investigate whether use of insulin pump therapy or continuous glucose monitor (CGM) alleviates FOH and increases physical activity levels in youth with T1D. The observational study revealed that CGM users had increased physical activity levels. However, they note that this was also the group that had frequent hypoglycaemic episodes (at least one per week), suggesting that a combination of modern diabetes management technique like CGM & high hypoglycaemia avoidant behaviour needs to be considered to mitigate exercise risk.

Lysy et al. trialled an algorithm-based insulin dose and carbohydrate intake adjusted exercise protocol for adolescents with T1D. Following a monitored first exercise session in the outpatient clinic to study participants’ glucose and insulin response during and after exercise, algorithm-based adaptions were suggested to patients for insulin doses and carbohydrate intake for the next session. The second session being a monitored real-life sport that the participant usually engages in. It was revealed that application of the algorithmic-based adaptations stabilized glucose excursions up to 15 hours after exercise. While the authors suggest this application could be incorporated in the clinical follow up of children and adolescents with T1D engaging in sport, further research is needed given the small sample size.

Co-prescription of exercise and diabetes medication is routine, often assuming additive or complementary benefits. Contradicting this is a recent review by Brinkmann. The comprehensive review offers a word of caution while prescribing non-insulin glucose-lowering class of medications like SGLT2 (Sodium-glucose cotransporter 2) inhibitors and exercise, concurrently. Which is similar to some, but not all, research on Metformin and exercise. Here, the attenuating effects of glucose lowering action of exercise and risk of ketoacidosis were cautioned with combined SGLT2 and exercise. The author suggests further research on tailoring exercise type to medications prescribed and vice versa to be the way forward to maximise the actions of medications and exercise.

Together, these articles offer new insight into the complexities and advancements in exercise as an adjunct management of diabetes; which is overlooked in terms of research input or knowledge acquisition in the healthcare sector. Given these are all preliminary studies which highlight the potential of such therapies/techniques, future research is needed. Specifically, to aid in developing guidelines for future translation into primary care, more details on the required frequency and intensity of Yoga-based lifestyle prescriptions and larger randomised controlled trials are needed. In addition, with the continual development of new pharmacological therapies for the treatment of type 2 diabetes research investigating the interactions of multiple medications (given many individuals are prescribed more than one agent) with traditional lifestyle therapies (diet and exercise) requires continual reviewing and consideration. 

## Author contributions

TC and MF drafted the paper, TC, BH and MF reviewed and finalized the manuscript. All authors contributed to the article and approved the submitted version.

## Conflict of interest

BH was employed by mediStatistica.

The remaining authors declare that the research was conducted in the absence of any commercial or financial relationships that could be construed as a potential conflict of interest.

## Publisher’s note

All claims expressed in this article are solely those of the authors and do not necessarily represent those of their affiliated organizations, or those of the publisher, the editors and the reviewers. Any product that may be evaluated in this article, or claim that may be made by its manufacturer, is not guaranteed or endorsed by the publisher.
